# Pregnancy with Persistent Hymen

**Published:** 1898-11-26

**Authors:** 


### PREGNANCY WITH PERSISTENT HYMEN.

Two cases have recently been published,[1] which are of some medico-legal as well as clinical interest, in both of which pregnancy took place notwithstanding the persistence of a hymen, the orifice in which was, indeed, much smaller than the normal. The first case was that of a young woman whom Dr. Cullinan attended in her first labour. On examining her he found the hymen "intact, except for a small opening which would allow the escape of the menstrual fluid, situated in the antero-posterior line about one-third from the anterior and two-thirds from the posterior edge of the vaginal opening, the aperture being of sufficient size to admit an ordinary slate pencil." The membrane was very tender and resistent, so it was incised by means of a hernia knife, after which labour proceeded naturally. The patient had been married eleven months.

The next case is that of a young woman brought to Dr. Harper Wigham complaining of being "irregular" and of having "the whites." She was not at first examined locally, but on this being done it was found that she had an offensive discharge, and on attempting to make a digital vaginal examination an obstruction was met with, "an extremely tough and dense hymen . . . . with a great convexity downwards and outwards, and with a feeling of resistance as if something solid existed beyond. There was found to be one small opening in the membrane in the anterior third just below the pubes," and on chloroform being given and the opening being forcibly enlarged, it was found that the mass above was a fœtus of about the fourth month with cord and placenta, all lying together in a globular dilatation of the upper part of the vagina. On these being removed it was found that the os was closed, and that the uterus was well contracted down. Apparently the condition had existed over a month at least. The patient had, in fact, aborted into her vagina, which was closed below by a persistent hymen with but a very small orifice.

